# Inter-Individual Budburst Variation in *Fagus sylvatica* Is Driven by Warming Rate

**DOI:** 10.3389/fpls.2022.853521

**Published:** 2022-04-13

**Authors:** Andrey V. Malyshev, Ernst van der Maaten, Aron Garthen, Dennis Maß, Matthias Schwabe, Juergen Kreyling

**Affiliations:** ^1^Experimental Plant Ecology, University of Greifswald, Greifswald, Germany; ^2^Chair of Forest Growth and Woody Biomass Production, TU Dresden, Dresden, Germany; ^3^Nationalparkamt Müritz, Hohenzieritz, Germany

**Keywords:** spring phenology, repeated phenological ranking, micro-site, tree morphogy, within-population variation, *Fagus sylvatica*, warming rate, bud dormancy

## Abstract

The onset of the growing season in temperate forests is relevant for forest ecology and biogeochemistry and is known to occur earlier with climate change. Variation in tree phenology among individual trees of the same stand and species, however, is not well understood. Yet, natural selection acts on this inter-individual variation, which consequently affects the adaptive potential to ongoing environmental changes. Budburst dates of 146 mature individuals of *Fagus sylvatica*, the dominant natural forest tree of central Europe, were recorded over 12 years in one forest stand of 1 ha in the Müritz National Park, Germany. The tree-specific location, topographical differences, as well as social status, were measured to explain the inter-individual variation in budburst. Furthermore, inter-individual differences in bud dormancy were quantified. Additional phenology and weather data across Germany from 405 sites over a 25-year period was used to put the insights from the single stand into perspective. Consistent phenological ranking over the years with respect to early and late flushing trees was observed within the single forest stand, with 23 trees consistently flushing 3–6 days earlier and 22 trees consistently flushing 3–10 days later than the median. Trees flushing consistently early varied most in their spring budburst dates and were less dormant than late-flushing trees already in mid-winter. The higher variation in earlier flushing trees was best explained by a slower warming rate during their budburst period in the observed stand as well as across Germany. Likewise, years with a lower warming rate during the budburst period were more variable in budburst dates. The rate of warming during spring time is crucial to accurately project future within-species variation and the resulting adaptive potential in spring phenology of dominant forest tree species.

## Introduction

Phenology plays a crucial role in the ecosystem’s carbon and water balances, and primary productivity (Kramer et al., 2000; Rötzer et al., 2004; Piao et al., 2008; Richardson et al., 2010). Phenological timing affects the survival, reproduction, and persistence of individuals and species (Larcher, 1981; Rathcke and Lacey, 1985) and therefore the distribution ranges of species (Chuine et al., 2010). Furthermore, the timing of spring phenology influences ecological interactions (Panchen et al., 2014), such as foraging and pollination (Hegland et al., 2009).

The variation in spring budburst timing between individual trees is crucial for the response of a population to environmental change. It results from the trade-off between an extended growing season and a higher risk of spring frost damage. Hence, inter-individual variation in spring phenology is a proxy for the selective pressure that an environment has on a species. Variation in tree phenology has been extensively studied at and above the species levels, i.e., comparing different populations within a species or comparing different species (Vitasse et al., 2009; Vitasse and Basler, 2013; Basler and Körner, 2014; Dantec et al., 2014; Marchin et al., 2015). Much less is known about phenological patterns at the population level, i.e., inter-individual variation (Cole and Sheldon, 2017; Delpierre et al., 2017). Knowledge about the intra-specific, inter-individual variation is relevant for understanding the adaptive potential of populations to proceeding climate change as well as for understanding the evolution and adaptation of populations at small geographical scales (Delpierre et al., 2017).

High phenological variability within a forest stand should theoretically make the stand more resilient against infrequent stressors such as late spring frost according to the portfolio effect (Markowitz, 1952) because the risk of frost damage is spread among a higher variation in leaf-out dates. Closed buds of our target species European beech are highly frost-tolerant (Kreyling et al., 2015), but even mild frost events damage recently flushed leaves. Leaves damaged by late frost are typically replaced, their production, however, requires resources otherwise used for wood production, leading to half or less of the usual radial growth in this species after late frost events (Príncipe et al., 2017). Late leaf-out reduces the risk of frost damage but shortens the growing season. Individuals leafing out late should consequently have advantages in spring frost years while individuals leafing out early should have advantages in non-frost years. Forest stands with a high variation in leaf-out timing thus possess biological bet-hedging (Cohen, 1966) and the potential for coping quickly with climate change.

Little is known about the causes of variation in budburst within single forest stands, even though phenological ranks in trees have been shown to repeat among individual trees, with inter-individual variation reaching three weeks over a 5-year period in numerous populations in common European trees (Delpierre et al., 2017). Observations stemming from 11 individuals of beech, all growing in the same botanical garden, suggests relevant and non-random inter-individual variation, spanning three weeks in the spring (Zohner et al., 2018). Such variation should potentially result in a competitive growth advantage of specific trees, depending on the weather conditions of any given year. The underlying mechanisms explaining variation on an individual level have rarely been studied. There is evidence, however, that warmer temperature during the budburst period decreases budburst variation (Denéchère et al., 2021). Possible environmental explanations are micro-meteorological variations in chilling temperatures, forcing temperatures, and photoperiod, i.e., the main drivers of spring phenology (Delpierre et al., 2017). However, photoperiod does not differ on a small geographical scale and dominant trees in given beech stands experience very similar temperatures as well (Capdevielle-Vargas et al., 2015; Gressler et al., 2015). Other tree-specific characteristics potentially affecting tree phenology include the tree social status (Gressler et al., 2015) and ontogeny (Vitasse, 2013). The variation in budburst timing between individuals not explainable by micro-environmental variation in driving factors, social status, and age is likely based on individual genetic differences (Bontemps et al., 2016). It has, for instance, been shown that individual trees can vary considerably in their photoperiod sensitivity, leading to differentiation in budburst even under the same environmental conditions (Zohner et al., 2018).

Microclimate differences and genotype do not sufficiently explain the variation in leaf unfolding date among individual trees (Fu et al., 2013). However, individuals may differ in their temperature sensitivity, just as they differ in photoperiod sensitivity (Zohner et al., 2018). Temperature sensitivity is generally quantified in warming sums that accumulate in the spring. The rate of warming sum accumulation differs from year to year and can then influence within-species variation in budburst dates, as evidenced by warmer spring temperature during the budburst period decreasing within-species variation in several tree species (Denéchère et al., 2021). After endodormancy (the inability of buds to open under optimal conditions) is released following a cold period, rapid warming in the spring should decrease budburst variation among trees whereas slow warming should increase budburst variation.

To explore the patterns and drivers of variation in budburst among individuals of the same forest stand, we analysed the spring phenology data for the dominant tree species of central Europe, *Fagus sylvatica* L. Leaf unfolding dates for 146 individual canopy trees from an old-growth stand in northeastern Germany were collected over 12 years. Publicly available phenological data were also analysed from 405 sites in Germany over a 25-year period. We quantified proxies for environmental differentiation and social status for each tree, with the aim to explain inter-individual and inter-annual variation in the timing of leaf unfolding. We aimed to determine the main factors influencing inter-individual budburst variation in a widespread tree species. We hypothesized that (1) differences in budburst dates between individual trees within a stand of mature beech trees would repeat across years due to differences in microsite and individual tree characteristics, that (2) budburst variation would be higher for trees that consistently flush during periods of slower warming rates in early spring, and, finally, we expected (3) higher variation in budburst dates in years with lower rates of temperature increase during the leaf flushing on small (forest stand) as well as on larger (countrywide) scales.

## Materials and Methods

### Study Area

The study area is situated in the beech forest district Serrahn Unit 5409 in Müritz National Park, Germany (53.34°N, 13.21°E). The site is characterized by a suboceanic-subcontinental climate, influenced by the temperate, humid-warm Atlantic climate and by the continental climate of Eastern Europe. The mean annual temperature in Serrahn is 7.9°C and the annual precipitation sum 627 mm (1951–1980). The site is located on the moraines of the Weichsel glaciation. The study area is situated about 100 m above sea level and has an undulating surface with plane areas and slopes between 7° and 24°. The main soil types are dystric cambisols, podzoluvisols, and luvisols, developed on loamy sand, and the dominant humus type is moder (Pohlers, 2011; Schröter et al., 2012).

The beech forest of Serrahn is characterized by a long period with little to no human intervention. A continuous forest cover was present since the 16th century. From 1849 to 1945, the study area was part of a game park. During this time, intensive forestry was prohibited and old-growth forests could be preserved. In the 1950s, parts of the forest, including the study area, were designated as a protected area and total reserve. In 1990, Serrahn became part of the Müritz National Park and in 2011 an area of 268 hectares was designated as a UNESCO World Natural Heritage site. Due to the long period of natural development, the biggest part of the forest is currently in the terminal phase of forest development and some parts are already in the degradation and regeneration phase.

### Phenological Data Collection

The Phenological data were recorded in an area of one hectare for 146 canopy trees of *Fagus sylvatica*. Each tree was marked with an individual number. The timing of leaf unfolding was monitored for each individual from 2008 to 2019. Spring budburst was defined as the first day when more than 50% of buds on a tree had open buds over the entire crown, with the leaves being in the coiled phase (not extending). To determine the stage of leaf development, binoculars were used. Each tree was observed every second day. Precision was ensured by observations being made predominantly by the same person, with new observers being initially accompanied by previous personnel.

### Climate Data

Daily temperature data were obtained from the German weather service (DWD) for the climate station Feldberg/Mecklenburg situated 15 km east of the study site. On-site temperature data, approximately 300 m from the site, had extended gaps, with the correlation between the temperature values of both sites giving an *R*^2^ of 0.95 (*F* = 9.96 * 10^5^; *p* < 0.001; *n* = 48266).

### Phenological Groups

**FIGURE 1:**
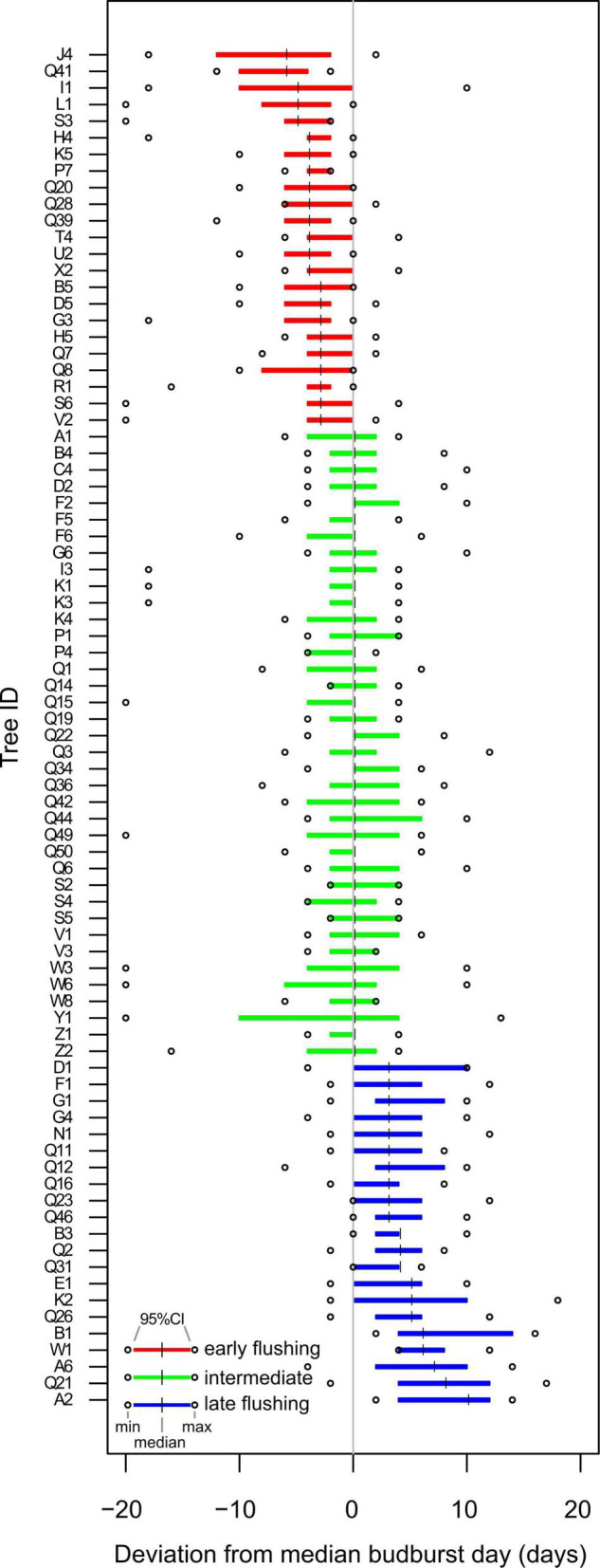
Temporally consistent phenological groups in a beech forest in Serrahn. All observed trees and their respective median deviation from the median tree across the 12 years of observations. Colors indicate the three phenological groups. Displayed are 95% CIs of the median deviation of each tree from the median tree. Dots represent tree-specific minimum and maximum median deviations across the 12 years.

If the deviation from the median budburst date for a given tree was earlier (smaller) than the median across all trees (=0 in Figure 1) over the 12 years of observation and the 95% CI did not exceed zero, the tree was termed “early”; if the median for a given tree was later (greater) than the median across all trees (=0 in Figure 1) and the 95% CI did not exceed zero, the tree was termed “late”; if the median for a given tree equalled the median over all trees (0 in Figure 1) and the 95% CI extended to positive and negative deviation, the tree was termed “intermediate.”

### Proxies of Environmental Conditions and Social Status

The micro-topographic setting was characterized for each tree, classifying the exposition at each tree over a radius of approximately 10 m in the three classes (1) bottom, (2) slope, and top (3). The spatial position of each tree was quantified by distance and angle to known positions (VERTEX IV-GS, Haglöf, Sweden). Elevation above sea level was determined from the spatial position and a Digital Elevation Model.

In 2018, the tree height and diameter at breast height (DBH) were determined for all individuals. Each tree was sorted into one of the following three social classes (1) dominant; (2) co-dominant; (3) subordinate based on their canopy position in relation to their direct neighbors.

### Dormancy Depth Estimation

To test whether early and late flushing trees already differed in mid-winter bud dormancy, twig samples were collected from specific trees on 10 January 2019. Five trees with the consistently earliest budburst dates and five trees with consistently latest budburst dates (cf. “phenological groups”) were sampled for dormancy depth estimation. The total *n* for this analysis was limited due to time constraints, as canopy branches were acquired using a slingshot (Bigshot, Sherrill Inc, Greensboro, NC, United States). Tree branches from the top third of each tree canopy were cut down and transported in cooling boxes to climate chambers (LT-36VLX, Percival, Perry, IA, United States) within 5 h of sampling, where they were cut into twigs (originating from three branches per tree) and placed in plastic cups filled with deionised water. Each twig measured approximately 15 cm. A total of thirteen twigs were prepared per tree, with eight of the twigs having their terminal buds excised to speed up budburst (terminal buds hinder lateral budburst, often failing to open before twigs die). Such cuttings have been shown to be a good proxy of relative bud dormancy depth, or the amount of warming required to force budburst at a particular time (Vitasse et al., 2014; Primack et al., 2015). A 16-h photoperiod at approximately 100 μmol m^–2^ s^–1^, a constant temperature of 22°C, and a humidity between 50 and 80% were maintained to force budburst. Due to deep dormancy at the time of sampling resulting in not all twigs surviving to budburst, only the forcing requirements of the first two flushing twigs with terminal buds and the first two flushing twigs with lateral buds were used in the analysis to allow a fair comparison among trees (budburst data pooled and averaged per tree). One early flushing tree had too many twigs dying prematurely and was not used in the analysis, resulting in a final comparison of nine trees. Dormancy depth was quantified as the number of days required for budburst under these forcing conditions. The respective dormancy depth of each tree was then correlated with its warming sum required for budburst in 2019.

### Quantifying Temperature Sensitivity of Phenological Classes

Time periods were determined during which increasing temperature had significant advancing or delaying effects on budburst dates. All possible combinations of start and end date from September 1 to the latest budburst date (May 10) were used to find the time period during which temperature best correlated with budburst dates by a Climate Window Analysis in R (Bailey and De Pol, 2016). The analysis was run separately for the early, intermediate, and late flushing trees to test the possibility that different optimal time periods exist for different trees. Only periods longer than 1 week were considered for ecological relevancy. The mean temperature during the most influential periods was then regressed against budburst dates of trees as well as against Growing Degree Days (GDD) required for budburst for each tree. GDD required for budburst to occur for a particular tree and year does not depend on how fast GDD accumulates, whereas budburst date does, being earlier for a faster rate of fulfilling the warming requirement. GDD was calculated as the sum of all daily mean temperatures above 5°C from January to the day of the budburst for each tree. Calculating the GDD from other starting dates (November, December, February), as well as from 1–3 months before budburst was also tried, with the January starting date giving the best correlation between GDD and budburst date (DF = 1837; Nov→F = 414, *p* < 0.001, *R*^2^ = 0.18; Dec→*F* = 1077, *p* < 0.001, *R*^2^ = 0.37; Jan→*F* = 1444, *p* < 0.001, *R*^2^ = 0.44; Feb→*F* = 1377, *p* < 0.001, *R*^2^ = 0.43). The temperature in degrees Celsius rather than chilling unit sums (e.g., number of days below a threshold cool temperature) was correlated against the GDD sums from January 1st to budburst as specific chilling thresholds may exist for each phenological class.

### Calculating Warming Rate During the Start of Tree-Specific Flushing Periods

The mean daily GDD accumulation until the earliest budburst date of each tree was calculated from the Serrahn budburst and temperature data. The rate of GDD accumulation during the earliest budburst date was better correlated with tree-specific budburst variation compared to the tree-specific mean budburst dates. A power function was fitted to estimate the non-linear rate of daily GDD accumulation in spring, whereby the day of the year was regressed against the mean daily GDD sum across the 12 years (log-transformed least squares regression *n* = 53; *p* < 0.001; *R*^2^ = 0.89; Supplementary Figure 1).

### Calculating Warming Rate During Year-Specific Flushing Periods

The start of the flushing period for each year was chosen as the date which was one SD below the mean budburst date of all trees. One SD above and below the mean was chosen as it incorporates a major portion of observations, that is 68%. The GDD range should estimate the GDD range required for the budburst of the majority of trees while minimising the chance of outliers influencing the result (single trees causing extreme increases/decreases in the GDD range). Then, for each year, the number of days required for a set GDD sum to accumulate from the start of the flushing period was calculated. The set GDD sum, that is the mean GDD sum required to complete budburst for most trees across years was averaged across all years and sites (Germany data) and across all individuals (Serrahn data). It provides the amount of warming required for flushing of 68% of the trees and was used as one fixed number per dataset. The set GDD sum spanned two standard deviations, being one standard deviation above and below the flushing GDD mean across all years, calculated as an interval from: [(GDD_*mean*_ + GDD_*SD*_) to (GDD_*mean*_ - GDD_*SD*_)]. Standard deviation in GDD rather than in budburst date was chosen to estimate the flushing period due to GDD_*SD*_ not being correlated with the warming rate itself (mean daily GDD accumulation). An illustration of the method is provided in Supplemental information (Supplementary Figure 2).

### Comparing the Main Factors Determining Budburst Variation Locally and Country-Wide

The relationship between the earliest budburst dates of individual trees across the monitored periods and their respective budburst variation was tested using the same calculations for the forest trees in Serrahn and for all regularly monitored trees in Germany. For Germany, the budburst and temperature data were obtained from the German Weather Service (DWD) from 405 sites (one tree per site) from 1994 to 2019 (Refer to Supplementary Figure 3 for location coordinates). The stations and years were chosen to maximise the number of trees for which data without missing years and stations was available. Mean temperature across all stations was used to calculate the GDD sum during the budburst period of each year. The earliest budburst date of each tree was regressed against its budburst variation to evaluate whether earlier flushing trees were always more variable in their budburst date. Additionally, the yearly starting budburst date in all trees was regressed against the respective yearly budburst variation.

### Statistical Analyses

The correlation between tree-specific dormancy depth and GDD sums at budburst was determined for the selected trees in Serrahn. Linear least-squares regression was fitted, relating dormancy depth of trees in the winter of 2019 with the spring GDD sum required for the budburst of the same trees.

For both Serrahn and Germany datasets, the earliest Budburst date of each tree was regressed against the respective trees’ budburst variation for Serrahn as well as for all monitored trees across Germany. Mean temperature during the most influential time periods of each year was regressed against budburst date and GDD sum at budburst for each tree.

For Serrahn data, the three phenological groups were tested with an ANOVA, while the year was used as a grouping variable for the random intercept: [lmer deviation from median ∼ phenological group + (1| year)]. Data were rank-transformed. The earliest budburst date of each tree and warming rate at the earliest budburst date of each tree were regressed against tree-specific variation in budburst date. The start of the budburst period and the warming rate during the budburst period were regressed against annual bud burst variation. Linear mixed-effect models were used to test potential differences in regression slopes between temperature means and budburst dates/warming sums at budburst (GDD). The predictor variables were fall/winter or spring temperature yearly means as well as the phenological tree category. Tree ID was used as a grouping variable for the random intercept and an ANOVA was used to compare if there was an interaction between the phenological group and temperature factors. Bonferroni correction was used to correct for multiple testing involving pairwise comparisons of the differences with respect to the phenological categories.

For the trees in Serrahn, the importance of the explanatory variables “tree height”, “tree exposition”, “DBH”, “elevation above sea level”, “tree dominance”, as well as the spatial x and y coordinates in meters of each tree on the median phenological deviation of each tree from the overall median, was quantified by boosted regression trees (BRT; Elith et al., 2008). BRT fits additive regression models and surpasses common linear models due to their ability to handle different types of predictor variables and missing values while not requiring prior data transformation or elimination of outliers. More importantly, BRT can fit nonlinear relationships and deal well with interactions between predictors. Generally, BRT outperforms most classical modeling techniques in their predictive power (Elith et al., 2008). Before fitting BRT, a reduction in dimensionality by removal of collinear variables was applied (Dormann et al., 2013). All candidate explanatory parameters were tested for collinearity with each other using Spearman’s nonparametric correlation. Where pairs of variables were highly correlated (*r* > 0.7), a univariate generalized additive model (GAM) was fitted to the data using each highly correlated variable. In order to obtain less correlated variables, the variable of each pair that yielded the greater Akaike information criterion (AIC) value was omitted, resulting in a final set of variables for the model. BRT was fitted according to Elith et al. (2008), with the selection of the final model based on minimal estimated cross-validated deviance, obtained by setting the tree complexity to 2, the learning rate to 0.00001, and the bag fraction to 0.5. The cross-validated correlation is used to express the correlation between the set of explanatory variables and phenological deviation from the median tree. For each explanatory parameter, its relative importance in explained variance is provided.

Furthermore, for the Serrahn data, an Analysis of Similarity (ANOSIM) analysis (Clarke, 1993) was run to test for spatial aggregation of early, late, and intermediate flushing trees. *x* and *y* coordinates were used to calculate a pairwise distance matrix for all trees. ANOSIM tests whether two or more groups differ significantly based on distance matrices.

All statistical analyses were performed using the R version 4.0.2 (R Core Team, 2021). using the additional packages climwin, ggplot2, lme4 1.1-14, lubridate_1.7.4, lmerTest 3.1-0, mgcv 1.8-31, dismo 1.1-4, gbm 2.1.5, vegan 2.5-6, ggplot2 3.1.1 and plyr 1.8.4.

## Results

### Phenological Ranking

Based on the budburst dates of all Serrahn trees over the 12-year period, 23 trees flushed consistently earlier than the median tree. Within these early flushers, the median deviation from the median tree ranged from 3 to 6 days over the 12 years, with the strongest recorded deviation being 20 days earlier than the median tree (Figure 1). Intermediate flushing trees totalled 38 individuals. A total of 22 trees flushed consistently later than the median tree. Within these late flushers, the median deviation from the median tree ranged from 3 to 10 days over the 12 years with the latest recorded deviation being 16 days later than the median tree (Average DOY for early, intermediate and late trees: 108, 112, 117; linear mixed effect model comparing the means of phenological classes with year as a grouping variable for the random intercept: *p* < 0.001, *F* = 284, DenDF = 970; *post hoc* test: *p* < 0.001 for all pairwise comparisons; Figure 1).

### Influence of Proxies for Environmental Differentiation and Social Status on Budburst Date

**FIGURE 2:**
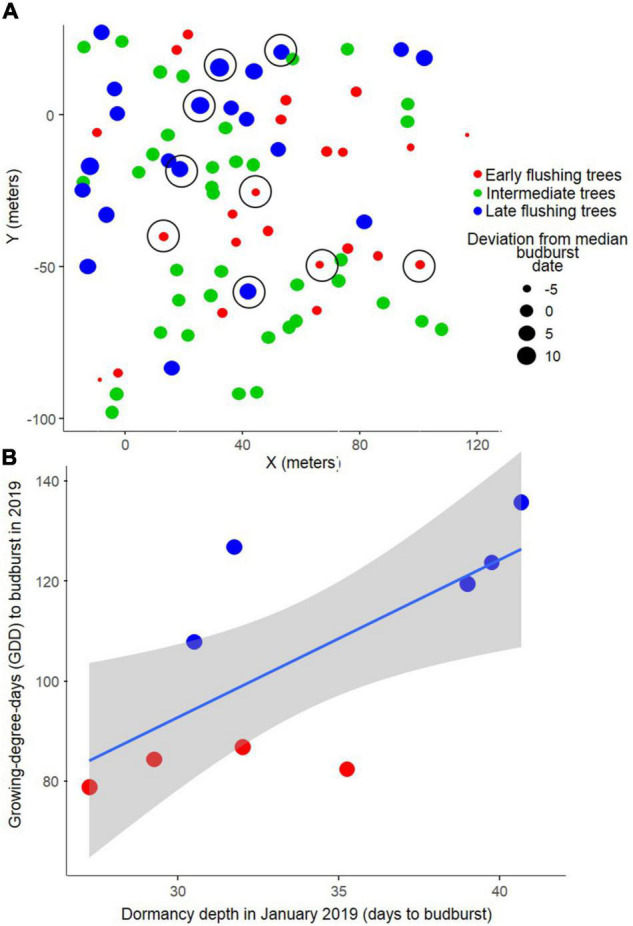
Earlier flushing beech trees are not spatially aggregated **(A)** and have lower bud dormancy depth already in mid-winter **(B)**. Late flushing trees (blue), intermediate trees (green), and early flushing trees (red), presented in terms of **(A)** their spatial arrangement, and **(B)** dormancy depth in winter regressed against the growing degree days (GDD) sums at budburst (from January 1) for the sampled trees in 2019. Five latest and four earliest trees were used (location of sampled trees marked by black circles in panel **(A)**. Grey polygons indicate 95% confidence bands.

For the Serrahn data, little to no explanatory power of the tested parameters on phenological deviation from the median in spring budburst dates was found by the Boosted Regression Tree analysis (cross-validated correlation *r*_*cv*_ = 0.13). The single parameters y-coordinate, elevation, tree height, DBH, x-coordinate, dominance, and exposition explained 36, 26, 15, 11, 6, 5, and 2%, respectively, of the low totally explained variance. The ANOSIM analysis did not show a significant spatial separation between the three phenological groups (Figure 2A; *R* = 0.02, *p* = 0.167).

### The Link Between Winter Bud Dormancy and Phenological Ranking

The deeper the January dormancy depth, the later was the budburst date of the sampled trees in 2019 (least-squares regression: *p* = 0.038; *R*^2^ = 0.41; *n* = 9), meaning that 41% of the variation in spring budburst variation in 2019 among the nine trees could be explained by winter dormancy depth about 4 months prior to budburst (Figure 2B).

### Budburst Variation Linked to Tree-Specific Budburst Dates

**FIGURE 3:**
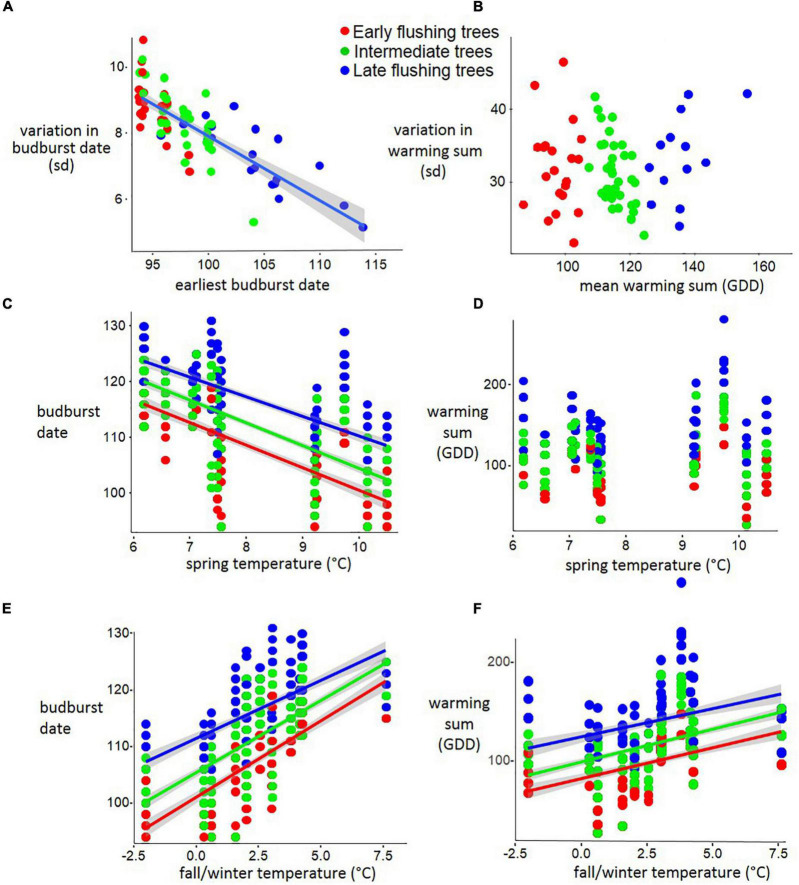
Earlier flushing beech trees have more variable budburst dates but are not more sensitive to temperature in a forest stand (Serrahn data). **(A)** The budburst date of each tree regressed against its variation across 12 years. **(B)** The mean warming sum (GDD from January 1) of each tree regressed against its variation in warming sum requirement across 12 years. **(C)** The budburst date of each tree regressed against the most influential spring temperature period (March 27 to April 27), also expressed as **(D)** GDD sums at bud break. **(E)** The budburst date of each tree regressed against the most influential fall/winter temperature period (November 22 to December 2), also expressed as **(F)** GDD sums at bud break.

In the forest stand of Serrahn, earlier flushing trees had more variable budburst dates (linear model: *p* < 0.001; *R*^2^ = 0.64; *n* = 81; Figure 3A) while not having more variable warming sum requirements (linear model: *p* < 0.55; n = 81; Figure 3B).

### Periods Influencing Budburst Timing

For the Serrahn data, the mean Temperature between March 27 to April 27 had the greatest advancing impact for all phenological classes (Linear models; AIC = –171 to –323; *R*^2^ = 0.47 to 0.51) while the temperature during Nov 21 to Dec 1 had the greatest delaying impact for all phenological classes (Linear models; AIC = –149 to – 363; *R*^2^ = 0.45 to 0.50). The correlations between temperatures during these periods and Budburst Dates and GDD sums are shown in Figure 3.

### Tree-Specific Temperature Sensitivity With Respect to Budburst Date and Warming Sums

For the Serrahn data, higher fall/winter temperature was related to later Spring Budburst (Early: *p* < 0.001, *R*^2^ = 0.53; β + 2.7; *n* = 276; Intermediate: *p* < 0.001, *R*^2^ = 0.52; β + 2.55; *n* = 456; Late: *p* < 0.001, *R*^2^ = 0.43; β + 2.04; *n* = 252; Figure 3E) and increased GDD sums at budburst (Early: *p* < 0.001, *R*^2^0.20; β + 6.3; *n* = 276; Intermediate: *p* < 0.001, *R*^2^ = 0.25; β + 6.68; *n* = 456; Late: *p* < 0.001, *R*^2^ = 0.43; β + 2.04; *n* = 252; Figure 3F). Fall/winter temperature affected earlier flushing trees stronger than later flushing trees as there was an interaction between the phenological category and rsvp temperature With respect to their effects on budburst date (early vs. mid *p* < 0.001; early vs. late *p* < 0.001; mid vs. late *p* < 0.001). Early flushing trees delayed their budburst the most after Warming fall/winter temperatures, followed by intermediate and then late flushing trees. Responses to spring temperature and fall/winter temperature in terms of warming sums at budburst (GDD) did not differ among the three phenological groups. Higher spring temperature (March 27 to April 27) was related to advanced spring budburst Dates at similar rates for early, intermediate and late trees (linear model: Early: *p* < 0.001; *R*^2^ = 0.47; β-4.08; *n* = 276; Intermediate: *p* < 0.001; *R*^2^ = 0.51; β−4.08; *n* = 456; Late: *p* < 0.001; *R*^2^ = 0.49; β−3.53; *n* = 252), while having no impact on GDD sums at budburst (Figures 3C,D).

### Effect of Warming Rate on Tree-Specific and Year-Specific Variation in Serrahn and Germany

**FIGURE 4:**
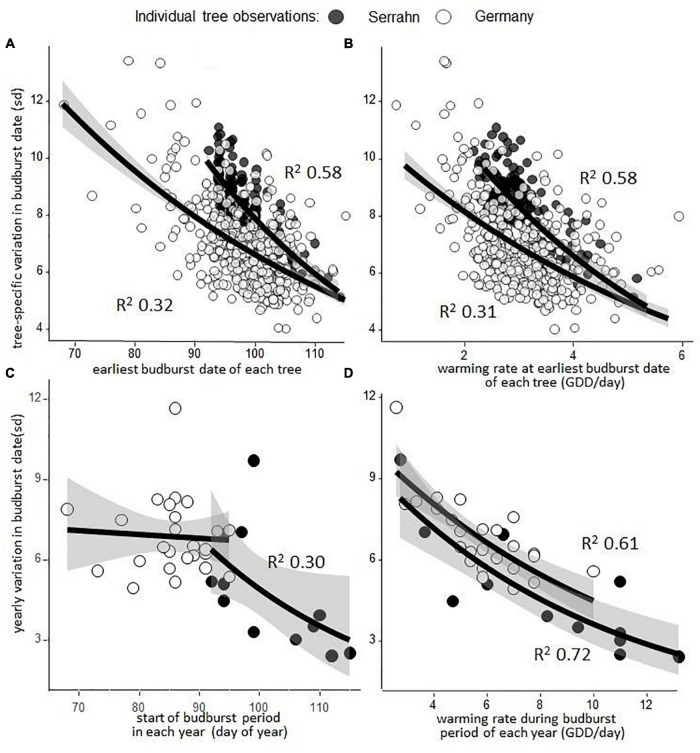
Tree-specific and year-specific variation in budburst dates is determined largely by the rate of warming during the budburst period. **(A)** The earliest budburst date of each of 146 trees in one forest stand (Serrahn) and of every single tree at 405 sites across Germany, regressed against the variation in budburst dates across the monitored 12 (Serrahn) or 25 (Germany) year periods, respectively. **(B)** The warming rate at the earliest budburst date for each monitored tree, quantified as mean GDD accumulation per day across the monitored years, regressed against the variation in budburst dates across the monitored 12 (Serrahn) or 25 (Germany) year periods, respectively. **(C)** The start of the budburst period in a forest stand (Serrahn) and across Germany in each year, regressed against the respective yearly variation in budburst date. **(D)** Warming sum accumulation rate in each year (GDD/day) in a forest stand (Serrahn) and across Germany during the budburst period, regressed against the respective yearly variation in budburst date. See section “Materials and Methods” for the budburst period definition.

Earlier flushing trees were more variable in their budburst dates than late flushing trees within one forest stand at Serrahn (power transformed linear regression: *p* < 0.001; *R*^2^ = 0.58; *n* = 159; Figure 4A) and across Germany (power transformed linear regression: *p* < 0.001; *R*^2^ = 0.32; *n* = 404; Figure 4A), due to the warming rate being slower during their starting budburst dates within one forest stand at Serrahn (power transformed linear regression: *p* < 0.001; *R*^2^ = 0.58; *n* = 159; Figure 4B) and across Germany (power transformed linear regression: *p* < 0.001; *R*^2^ = 0.31; *n* = 159; Figure 4B). Years with earlier starting budburst periods had higher budburst variation within one forest stand at Serrahn (power transformed linear regression: *p* < 0.04; *R*^2^ = 0.30; *n* = 12) but not across Germany (power transformed linear regression: *p* = 0.76; *n* = 25; Figure 4C). Years with slower warming rates during the budburst period had higher budburst variation within one forest stand at Serrahn (power transformed linear regression: *p* < 0.001; *R*^2^ = .72; *n* = 12) and across Germany (power transformed linear regression: *p* < 0.001; *R*^2^ = 0.61; *n* = 25; Figure 4D).

## Discussion

The single most consistent factor able to explain budburst variation among individuals of one forest stand as well as across many sites in Germany was the spring warming rate. Within the observed forest stand, consistently earlier flushing trees tended to open their buds when the rate of warming was slow, causing large differences in the number of days required to fulfill their warming requirements from year to year. Likewise, across Germany, a major part of yearly variation in budburst dates in *Fagus sylvatica* was attributable to the warming rate during the flushing period.

The variation in budburst dates between the single trees in a forest stand of only 1 ha area was similar to the variation of single trees from 405 sites with contrasting climatic conditions all across Germany (Figure 4) and close to the variation between twelve populations spanning approx. 10^°^ in longitude and latitude (Delpierre et al., 2017).

The presence of consistently early and consistently late flushing trees in a single forest stand is a novel finding. Previous observations from single stands report either no repeated ranking in individual tree budburst order (Capdevielle-Vargas et al., 2015) or show consistent ranking albeit in a much larger forest of 385 ha where micro-environmental differences are highly probable (Cole and Sheldon, 2017). Still, consistently repeating orders of budburst have also been observed between beech populations at sites across several hundred kilometers and, consequently, across contrasting environmental conditions (Delpierre et al., 2017). Repeated ranking in budburst dates of individual trees can potentially be explained by microclimate variation (Fu et al., 2014; Hwang et al., 2014; Delpierre et al., 2017) and/or tree social status and competition (Schieber, 2006). Dominant trees have been found to flush later than subordinate, co-dominant trees, although the consistency of such a pattern across years is not statistically supported (Schieber, 2006). Interestingly, social status, tree height, and DBH as proxies for competitive status or tree exposition, microrelief, and elevation as proxies for environmental differentiation did not explain a substantial part of the variation in spring phenology in our data. We even did not detect any spatial pattern which could be related to the observed inter-individual pattern in budburst. Chesnoiu et al. (2009) proposed spatial grouping of early and late flushing oak trees, although clustering was only visually interpreted for one year of observations. Repetition in spring phenology ranking between populations has been shown to be higher in sites with higher spring water content (Delpierre et al., 2017). Nonetheless, the effect of soil water content on spring budburst is not functionally established (Angus and Moncur, 1977; Penuelas et al., 2004; Schmull and Thomas, 2000), and soil water content in our target stand is expected to vary mainly with micro-relief, which did not provide substantial explanatory power in our analyses. Measurements of top-soil moisture of selected trees within one spring within the forest stand have also not been shown to correlate with budburst dates (data not shown). Thus, we hypothesize that consistent phenological ranking of leaf unfolding at our study site is potentially attributable to genetic differentiation (Crawley and Akhteruzzaman, 1988; Delpierre et al., 2017), independent of environmental factors.

Accordingly, earlier flushing trees were less dormant already in mid-winter, about 4 months before budburst, although due to the small sample size and low percentage of twigs surviving to budburst the result has to be interpreted with caution. Certain trees thus always require a lower warming sum to flush in spring, regardless of preceding weather conditions. It has been shown that populations within a species can differ in dormancy patterns under the same environmental conditions (Myking and Heide, 1995). Specifically for beech, the similar sensitivity of dormancy to temperature and photoperiod has been shown among populations (Heide, 1993; Malyshev et al., 2014), whereas individual trees can differ in photoperiod sensitivity (Zohner et al., 2018). Furthermore, a common garden trial has shown a clear influence of genetic differences of beech populations from different origins across Europe on spring phenology (Wuehlisch et al., 1995; Robson et al., 2013). Trees consistently flushing early are more likely to cross-pollinate with other early trees, as flowering occurs simultaneously with leaf out in beech. Consequently, phenological segregation may persist, ensuring that the resulting variation is preserved in a population until natural selection swings the pendulum into the direction of one or the other due to insect emergence, spring frost occurrence, or variable growing season lengths persistently, thereby reducing variation within the stand.

### Higher Temporal Variation in Budburst Dates in Early Flushing Trees Due to Higher Temperature Sensitivity

Earlier flushing trees were more variable in their spring budburst dates within a single forest stand as well as between sites across Germany (Figure 4). The differential photoperiod sensitivity of individual beech trees has been proposed to explain consistently earlier flush and larger variability of certain beech trees as compared to others (Zohner et al., 2018). We propose that the warming rate during the budburst period of each tree is an additional and, potentially, more influential factor explaining higher variation in spring phenology of consistently early-flushing trees. As a higher spring temperature sensitivity in budburst dates was not found for the warming sum needed for budburst (GDD), it is likely that the slower rate of warming during the budburst period of earlier flushing trees was the cause of the afore-mentioned differences and not due to earlier flushing trees having unique physiological responses. Due to their inherently low warming sum requirements (Figure 3F), early-flushing trees flush during times of slower rates of warming (Supplementary Figure 1). Budburst variation is, thus, largely dependent on trees with low warming requirements, making them the most variable in response to differences in spring warming rates between years (Denéchère et al., 2021) and largely determining the budburst variation on a local scale.

### Higher Variation in Budburst Dates in Years With Slower Spring Warming

The warming rate during the budburst period explained most of the variation in budburst dates across years in Germany. Denéchère et al. (2021) came to similar results albeit with fewer years of observation but for other tree species as well, showing that warmer temperature during budburst decreases within-species budburst variation. Year-to-year budburst periods can strongly overlap in their ranges while differing strongly in their rate of warming (Supplementary Figure 4). We argue therefore that predicting the simple, yet up to now overlooked parameter, warming rate during the budburst period (Denéchère et al., 2021) will most accurately be able to predict future budburst variation.

## Conclusion

Beech trees within a single forest stand differed strongly in their spring phenology and did so consistently over the years. Inherent differences in warming sum requirements are likely responsible for the consistent phenological ranking of the individual trees, with little influence from tree-specific positional and topographical differences. Lower dormancy depth of earlier flushing trees already in mid-winter results in such trees being more variable in their spring budburst due to a consistently lower rate of warming sum accumulation early in the spring. Projecting overall changes in annual variation of budburst dates in a species can be improved if models are able to project future warming rate changes during future bud bursting periods.

## Data Availability Statement

The raw data supporting the conclusions of this article will be made available by the authors, without undue reservation.

## Author Contributions

AM analysed the data and wrote the manuscript. AG and DM collected and compiled morphological and microsite characteristics of the beech trees. MS was in charge of phenological data collection. JK and EM helped to analysed the data and wrote the manuscript. All authors contributed to the article and approved the submitted version.

## Conflict of Interest

The authors declare that the research was conducted in the absence of any commercial or financial relationships that could be construed as a potential conflict of interest.

## Publisher’s Note

All claims expressed in this article are solely those of the authors and do not necessarily represent those of their affiliated organizations, or those of the publisher, the editors and the reviewers. Any product that may be evaluated in this article, or claim that may be made by its manufacturer, is not guaranteed or endorsed by the publisher.
