# Supplementary material for: Inter-Individual Budburst Variation in Fagus sylvatica Is Driven by Warming Rate
Source: Front Plant Sci. 2022 Apr 13;13:853521. doi: 10.3389/fpls.2022.853521 (PMC9044039; doi:10.3389/fpls.2022.853521)
Supplement: Supplementary file 1 [file Data_Sheet_1.PDF]

Supplements

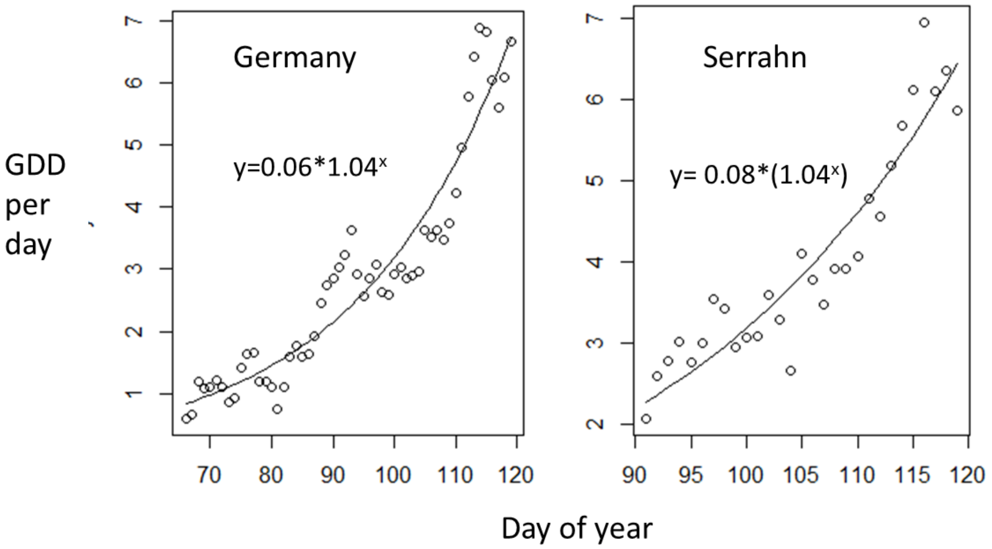

Figure S1. Power functions fitted to estimate the rate of daily GDD accumulation on each day of year in spring whereby day of year was regressed against daily GDD sum across the 12 years for Serrahn and across 25 years for Germany.

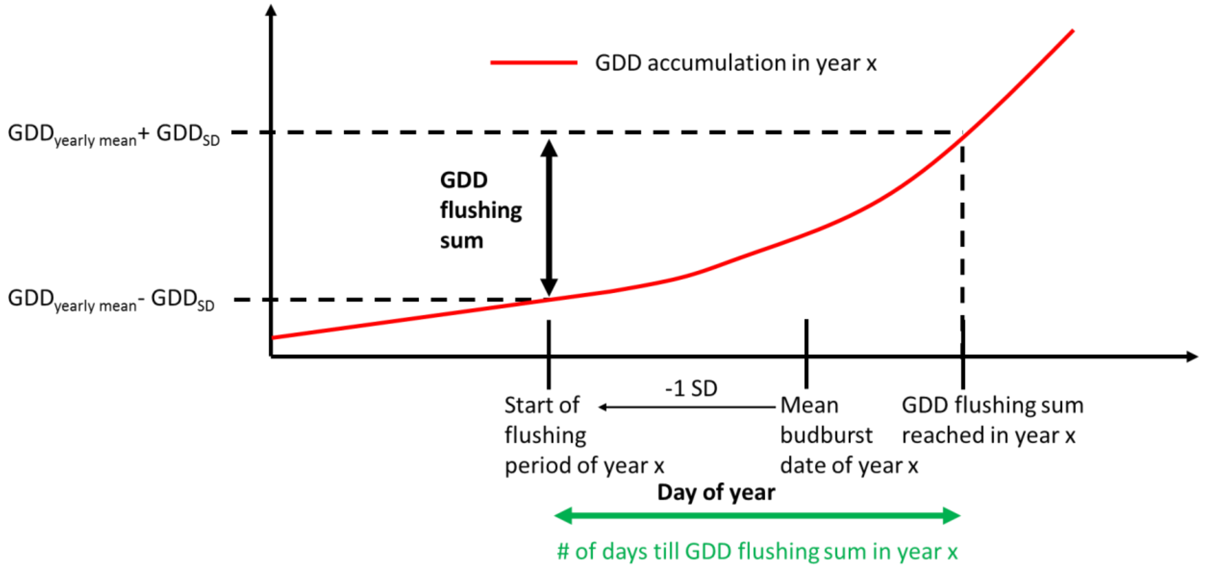

GDD flushing sum→ Mean GDD sum across years = $((GDD_{mean} + GDD_{SD}) - (GDD_{mean} - GDD_{SD}))$

Warming rate during flushing in year x (GDD day^{-1}) = GDD flushing sum / # of days till GDD flushing sum

Figure S2: Explanation of how the year-specific warming rate during budburst was calculated for the forest stand in Serrahn as well as across Germany. Tree-specific warming rate at the earliest budburst date of each tree was estimated using the power-fitted function of the spring temperature increase in Figure S1.

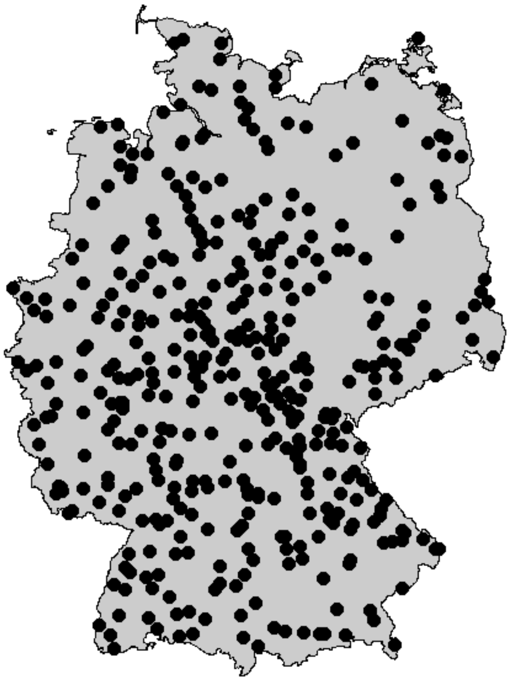

Figure 3: Locations of 405 monitored *Fagus sylvatica* trees across Germany used in the analysis of spring budburst variation from 1995 to 2019.

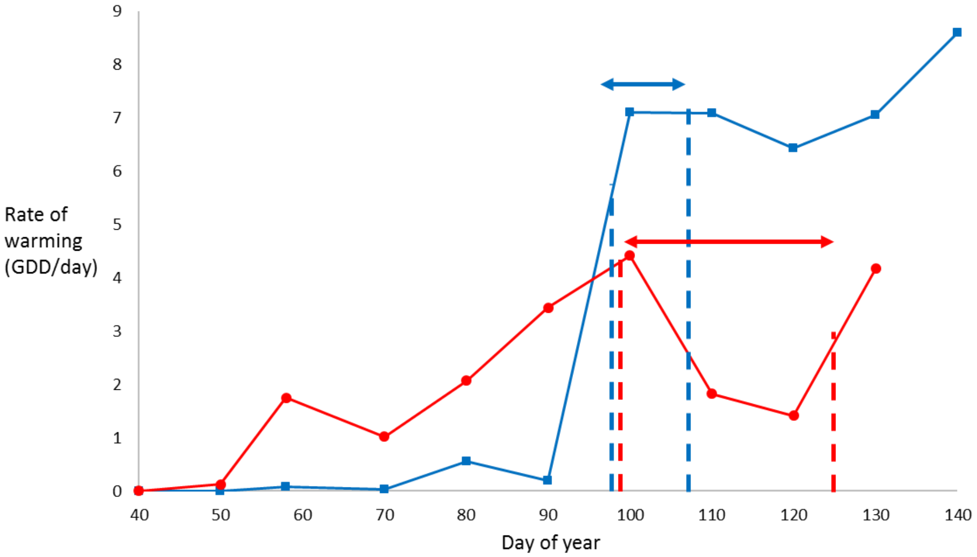

Figure S4: Yearly budburst variation depends on the rate of warming during budburst period and not on the date of budburst. Blue solid line: daily rate of warming accumulation (means of 10 consecutive days plotted) in year with lowest budburst variation in Germany across 405 sites (2009; s.d. +/- 4.9). Red solid line: year with highest budburst variation (2017; s.d. +/- 11.7). Dotted lines: start and end of each flushing period in each year (mean -/+ s.d.). Lines with arrows show the respective budburst variations.
